# The potential involvement of FABP4 in rheumatoid arthritis-associated osteoporosis: a comprehensive bioinformatics analysis and experimental validation

**DOI:** 10.3389/fimmu.2026.1738443

**Published:** 2026-05-28

**Authors:** Xu Tang, Na Liu

**Affiliations:** Department of Rheumatology and Immunology, Xinhua Hospital Affiliated to Dalian University, Dalian, China

**Keywords:** bioinformatics analysis, collagen-induced arthritis, fatty acid-binding protein 4, osteoporosis, rheumatoid arthritis

## Abstract

**Objective:**

This study investigates the role and underlying mechanism of FABP4 in RA secondary OP by examining its correlation with the condition.

**Methods:**

DEGs related to RA and OP were identified using GEO datasets (GSE17755 and GSE230665), focusing on bone metabolism genes. Candidate genes were identified using WGCNA, PPI networks, and four algorithms (Degree, BottleNeck, MCC, EPC), followed by enrichment analysis. Key genes were pinpointed using machine learning methods (SVM-RFE, LASSO, Boruta) and validated with ROC curves, all showing AUC > 0.7. MicroRNA prediction and genomic mapping were also done. For *in vivo* validation, CIA rats (n=5 per group) were analyzed at 2, 6, and 12 weeks against a control group using ELISA, micro-CT, H&E staining, IHC, and quantitative image analysis.

**Results:**

Bioinformatics identified eight hub genes: PTEN, CDC42, MFN2, UBC, RAS5F1, MMP9, CCL17, and FABP4. In CIA rats, FABP4, TNF-α, IL-6, IL-1β, RANKL, and β-CTX levels rose with disease progression, showing slight increases at 2 weeks and significant rises at 6 and 12 weeks (P<0.05). At 12 weeks, these levels were higher than at 6 weeks (P<0.01). FABP4 levels positively correlated with TNF-α, IL-6, IL-1β, RANKL, and β-CTX (P<0.01). Micro-CT showed worsening ankle joint damage over time. At 2 weeks, CIA rats had no significant BS/BV difference from controls but showed decreased BV/TV and Tb.Th and increased Tb.Sp (P<0.05). At 6 and 12 weeks, CIA rats had significantly lower BV/TV, BS/BV, and Tb.Th and higher Tb.Sp compared to controls (P<0.01). IHC analysis showed high FABP4, TNF-α, and RANKL expression in CIA rats’ synovium and cartilage at 2 weeks. TNF-α levels stabilized by 6 weeks and returned to normal by 12 weeks. FABP4 peaked at 12 weeks, while RANKL decreased but stayed above 2-week levels.

**Conclusion:**

FABP4 is closely associated with abnormal bone metabolism in RA secondary OP, potentially through the RANKL/RANK/OPG system. It may represent a potential therapeutic target that warrants further investigation.

## 1 Introduction

Rheumatoid arthritis (RA) is a systemic autoimmune disorder marked by persistent synovitis, joint destruction, and systemic inflammation, and it is a leading cause of joint deformity and functional disability (1, 2). Affecting approximately 1% of the global population, RA imposes a significant healthcare burden and often results in severe bone metabolic complications (3, 4). Individuals with RA frequently encounter the dual challenges of generalized osteoporosis and localized bone erosion, with the prevalence of secondary osteoporosis (OP) ranging from 22% to 36% (5), thereby further diminishing quality of life and elevating fracture risk. Traditional explanations for this bone loss include chronic inflammation-induced bone resorption, decreased physical activity, and glucocorticoid use. Nonetheless, emerging evidence indicates that these factors alone do not fully account for the severity and early onset of bone loss in RA, suggesting the existence of a systemically driven bone metabolic imbalance (6, 7). Consequently, a more profound understanding of the mechanisms linking inflammation to excessive osteoclast activity is essential for effective prevention and treatment strategies.

The disruption of bone immune homeostasis has garnered increasing scholarly attention in the context of RA-associated secondary OP. Among the pivotal mediators, the pronounced dysregulation of the RANKL/RANK/OPG signaling pathway serves as a central mechanism in the pathological bone resorption characteristic of RA-associated secondary OP (8). Receptor activator of nuclear factor kappa-B ligand (RANKL) interacts with its receptor, RANK, thereby stimulating osteoclast activity (9). In patients with RA, fibroblast-like synoviocytes (FLS) exhibit elevated levels of RANKL, which directly activate osteoclast precursors (10). Activated T lymphocytes secrete RANKL and induce synovial fibroblasts to upregulate RANKL expression, thus facilitating osteoclastogenesis, while B cells enhance RANKL expression through Fc receptor γ-chain signaling activated by autoantibodies (11). Elevated levels of pro-inflammatory cytokines, such as tumor necrosis factor-alpha (TNF-α), Interleukin (IL)-1β, and IL-6, further exacerbate the imbalance in bone metabolism. TNF-α activates the NFATc1 pathway and induces elevated RANKL expression in synovial fibroblasts (12). IL-1β acts synergistically with RANK to reduce the threshold for osteoclast differentiation and inhibits collagen synthesis. IL-6 contributes to osteoclastogenesis and induces ferroptosis in osteoblasts (13, 14). Osteoprotegerin (OPG) functions as a decoy receptor for RANKL, thereby inhibiting osteoclast differentiation (15). Inflammatory cytokines downregulate OPG transcription via activation of the nuclear factor kappa-light-chain-enhancer of activated B cells (NF-κB) signaling pathway, while the RANK/OPG complex depletes OPG, thereby exacerbating bone resorption (16). In patients with RA, elevated RANKL/OPG ratios in serum and synovial fluid are strongly associated with increased bone erosion scores and reduced bone mineral density (BMD) (17). Therefore, targeting the intra-articular immune interaction network that facilitates bone destruction emerges as a promising therapeutic approach for RA.

Fatty acid-binding protein 4 (FABP4), a critical member of the fatty acid-binding protein family, is secreted by macrophages and adipocytes. It plays a regulatory role in inflammation, promotes cell proliferation and angiogenesis, and is implicated in cancer and immunometabolic disorders. Inhibition of FABP4 in renal tubular epithelial cells significantly decreases the levels of inflammatory cytokines such as IL-6, MCP-1, and TNF-α (18). Conversely, the administration of exogenous FABP4 enhances the expression of these inflammatory mediators in the Achilles tendon (19). In RA, increased expression of FABP4 is positively correlated with disease severity (20). FABP4, secreted by M1-polarized macrophages within RA synovial tissue, activates the NF-κB signaling pathway, thereby promoting the secretion of inflammatory cytokines by endothelial cells and synoviocytes (21). This activation enhances cellular proliferation, migration, invasion, and cytokine production, facilitates angiogenesis, and disrupts chondrocyte homeostasis. Furthermore, both serum and synovial fluid levels of FABP4 are markedly elevated in RA patients compared to those with osteoarthritis (22). Recent research indicates that FABP4, through the formation of a fabkin complex, modulates RANKL-mediated mitogen-activated protein kinase (MAPK) and NF-κB pathways, promoting osteoclast maturation and bone resorption, thereby exacerbating the pathological progression of OP (23). Nonetheless, the interaction between FABP4 and RANKL in RA-associated secondary OP remains insufficiently understood.

In this study, we utilized bioinformatics analyses to investigate the correlation between the FABP4 gene and the RANK/RANKL/OPG signaling pathway. Subsequently, we conducted *in vivo* experiments to evaluate inflammatory markers and the levels of FABP4 and RANKL in the serum and ankle joints of RA model rat. Our aim is to provide exploratory insights into the possible molecular mechanisms underlying RA-associated secondary OP and to identify potential biomarkers and therapeutic targets for clinical diagnosis and treatment.

## 2 Materials and methods

### 2.1 Data collection

The datasets used in this study include GSE17755 (rheumatoid arthritis, RA: 112 cases; control: 45 cases; synovial tissue/synovial fibroblasts) and GSE230665 (osteoporosis, OP: 12 cases; control: 3 cases; peripheral blood/whole blood). Both datasets were obtained from the GEO database (https://www.ncbi.nlm.nih.gov/geo/). Genes related to bone metabolism were derived from the KEGG database (https://www.genome.jp/kegg/). Specifically, all annotated genes of the RANK/RANKL/OPG signaling pathway (pathway ID: hsa04380) were extracted. After removing duplicates, 143 bone metabolism−specific genes were obtained for subsequent analyses.

### 2.2 Differential gene expression analysis

Differential gene expression analysis was conducted on the GEO datasets GSE17755 (rheumatoid arthritis, RA) and GSE230665 (osteoporosis, OP). Differentially expressed genes (DEGs) were identified using the “limma” R package (v3.54.0) with the thresholds of an adjusted p-value < 0.05 and an absolute log2 fold change (|log2FC|) > 1. Visualization of the DEGs was achieved through a heatmap generated with the “Complex Heatmap” package (v2.15.1) and volcano plots created with “ggplot2” (v3.4.1). The resulting DEGs were subsequently used for further analysis to delineate disease-specific expression profiles.

### 2.3 WGCNA and identification of candidate genes

Weighted gene co-expression network analysis (WGCNA; v1.72.0) was performed on the merged dataset to identify co-expression modules. Hierarchical clustering based on Euclidean distance revealed no outlier samples. A soft threshold was determined to fit a scale-free topology, followed by module identification. Genes with high module membership in key modules of interest were selected as bone metabolism-related module genes. Subsequently, the overlap between these module genes, RA-related differentially expressed genes (RA-DEGs), and OP-related DEGs (OP-DEGs) was taken. This triple intersection, visualized using the “VennDiagram” package (v1.7.1), defined the final set of candidate genes for this study.

### 2.4 PPI network construction and identification of key genes

Protein-protein interaction (PPI) network was built using the STRING online database, with interactions filtered at a medium confidence score >0.4. Candidate genes were ranked using four algorithms (Degree, BottleNeck, MCC, and EPC), and the top 20 genes from each ranking were selected. The intersection of these selections yielded hub genes.

### 2.5 Enrichment analysis

GO and KEGG pathway analyses were performed on the candidate genes utilizing the R package “clusterProfiler(p-value<0.05)”. GO annotation enrichment terms are determined based on biological processes (BP), cellular components (CC), and molecular functions (MF) to identify their biological characteristics. KEGG enrichment analysis is also performed to identify key signaling pathways associated with candidate genes.

### 2.6 Hub gene identification and diagnostic validation

To refine the key genes from the network, we employed three distinct machine learning algorithms: Support Vector Machine-Recursive Feature Elimination (SVM-RFE), LASSO (Least Absolute Shrinkage and Selection Operator) regression, and the Boruta algorithm. The intersection of genes identified by these three methods was defined as the final diagnostic biomarkers. Their discriminatory power between patient and control samples was evaluated by generating Receiver Operating Characteristic (ROC) curves for each gene using the pROC R package, and the Wilcoxon Rank Sum Test was applied to assess significance.

### 2.7 Genomic localization and miRNA prediction of hub genes

The chromosomal locations of the eight candidate genes were determined. Use mirdb (https://mirdb.org/), mirnada (http://www.bioinformatics.com.cn/local_miranda_miRNA_target_prediction_120) and microcosm (https://mycocosm.jgi.doe.gov/) three separate forecast targeting key candidate database Potential mirna of genes. To improve the specificity of the prediction, the results from the three databases were intersected.

### 2.8 Establishment and grouping of the CIA model rats

Twenty Sprague-Dawley (SD) rats (aged 7–8weeks, weighing 200 ± 20g) were purchased from LIAONING CHANGSHENG BIOSCIENCE Co, and housed in the Laboratory Animal Center of School of Clinical Medicine of Xinhua, Dalian University with a temperature between 22 and 24 °C, humidity between 55% ~60%, and light/dark cycle for 12h. All the rats could get food and water freely. The protocols of this test were approved by the Experimental Animal Ethics Committee (2023-167-01). Five rats were randomly selected as the control group (Control): injection of saline only. CCII (Sigma Aldrich) at a concentration of 2 mg/mL was fully emulsified with an equal volume of CFA (Sigma Aldrich). A 0.2mL aliquot of this emulsion was intradermally injected into each of the rats’ rear footpads. The day of the first injection was defined as day 0. Seven days later, the rats received a booster injection: an equal volume of CCII emulsion was injected into the same site (24). On Day14, fifteen rats with an arthritis index ≥4 were regarded as collagen-induced arthritis (CIA) models which were randomly divided into 3 groups: 2w (n=5), 6w (n=5), and 12w (n=5).

### 2.9 Ethology

Starting from the initial immunization (Week 0), the thickness of the plantar pads of both hind paws of rats at the same position was measured weekly using a vernier caliper, and the average value was calculated. Body weight was recorded every 7 days. The arthritis index score (AI score) was utilized weekly to evaluate the severity of arthritis by visual inspection, which was estimated and recorded according to the swelling and activity of joints with a cumulative score of the severity of arthritis in 4 limbs. The total scale score ranged from 0 to 16, with the scoring criteria as follows:

0 points: no joint swelling observed;

1 Point: slight swelling of the small phalangeal joint;

2 Points: moderate swelling of the phalangeal joint and toes;

3 Points: total swelling of the phalangeal joint and toes below the ankle joint;

4 Points: total foot swelling including the ankle joint (25).

### 2.10 Elisa

Blood samples were collected via cardiac puncture and centrifuged at 3000 rpm for 15 minutes to isolate serum. The serum levels of FABP4, TNF-α, IL-6, IL-1β, RANKL, and β-CTX were quantified using commercial enzyme-linked immunosorbent assay (ELISA) kits according to the manufacturer’s instructions. Briefly, standards and samples were added to antibody-coated wells and incubated at 37 °C for 30 min. After washing, avidin-biotin-peroxidase complex (ABC) working solution was added and incubated. Following another wash, tetramethylbenzidine (TMB) was added and the reaction was stopped with stop solution after 15 minutes. Finally, the absorbance was measured at 450 nm by an ELISA reader.

### 2.11 Micro-CT analysis

Hind paws were scanned using a micro-CT system with the following parameters: voltage of 80kv, current of 1mA,2000 projections, exposure time 56ms, and resolution of 60um. Three-dimensional reconstructions were generated from the acquired images. The calcaneus was selected as the region of interest (ROI) for the quantitative analysis of the bone microstructure, including the bone volume/tissue volume (BV/TV), bone surface/volume (BS/BV), trabecular thickness (Tb. Th), trabecular separation (Tb. Sp).

### 2.12 H&E staining

Hind paw tissues was fixed in 4% paraformaldehyde for 48h and decalcified in 0. 5M EDTA with pH 7. 2. The ankle joints were then dehydrated, embedded in paraffin, and sectioned at 4–5μm thickness. Sections were deparaffinized, rehydrated, and stained with hematoxylin and eosin (H&E) to evaluate synovial hyperplasia and inflammatory cell infiltration in the joint tissue.

### 2.13 Immunohistochemistry

Tissue sections were deparaffinized, rehydrated, and subjected to antigen retrieval. Endogenous peroxidase activity was quenched with 3% H_2_O_2_. Sections were incubated overnight at 4°C with primary antibodies against FABP4, RANKL, and TNF-α. After washing, the sections were incubated with a horseradish peroxidase (HRP)-conjugated secondary antibody at 37°C for 20 min. Signal was developed using a 3,3′-diaminobenzidine (DAB) kit, and nuclei were counterstained with hematoxylin. Stained sections were imaged under a light microscope for analysis.

### 2.14 Statistical analysis

For the analysis of animal specimen data, SPSS version 27.0 software was employed to assess normality and homogeneity of variance in the measurement data. In instances where the data conformed to a normal distribution, a one-way analysis of variance (ANOVA) was utilized for comparisons among multiple samples, while an independent samples t-test was applied for comparisons between two samples. The measurement data were reported as mean ± standard deviation (x ± s). Initially, a homogeneity of variance test was conducted. If the variance was homogeneous, Fisher’s Least Significant Difference (LSD) test was employed; in cases of heterogeneous variance, Tamhane’s T2(M) test was used for pairwise comparisons. For correlation analysis, Pearson correlation analysis was conducted when the data followed a normal distribution; otherwise, Spearman correlation analysis was applied. A p-value of less than 0.05 was considered indicative of statistical significance. Quantitative IHC was assessed using ImageJ (NIH, USA). Three random high-power fields (×400) per section were selected, and the histochemical score (H−score) was calculated as the percentage of positively stained area.

## 3 Results

### 3.1 Gene selection process and differential analysis results

**Figure 1:**
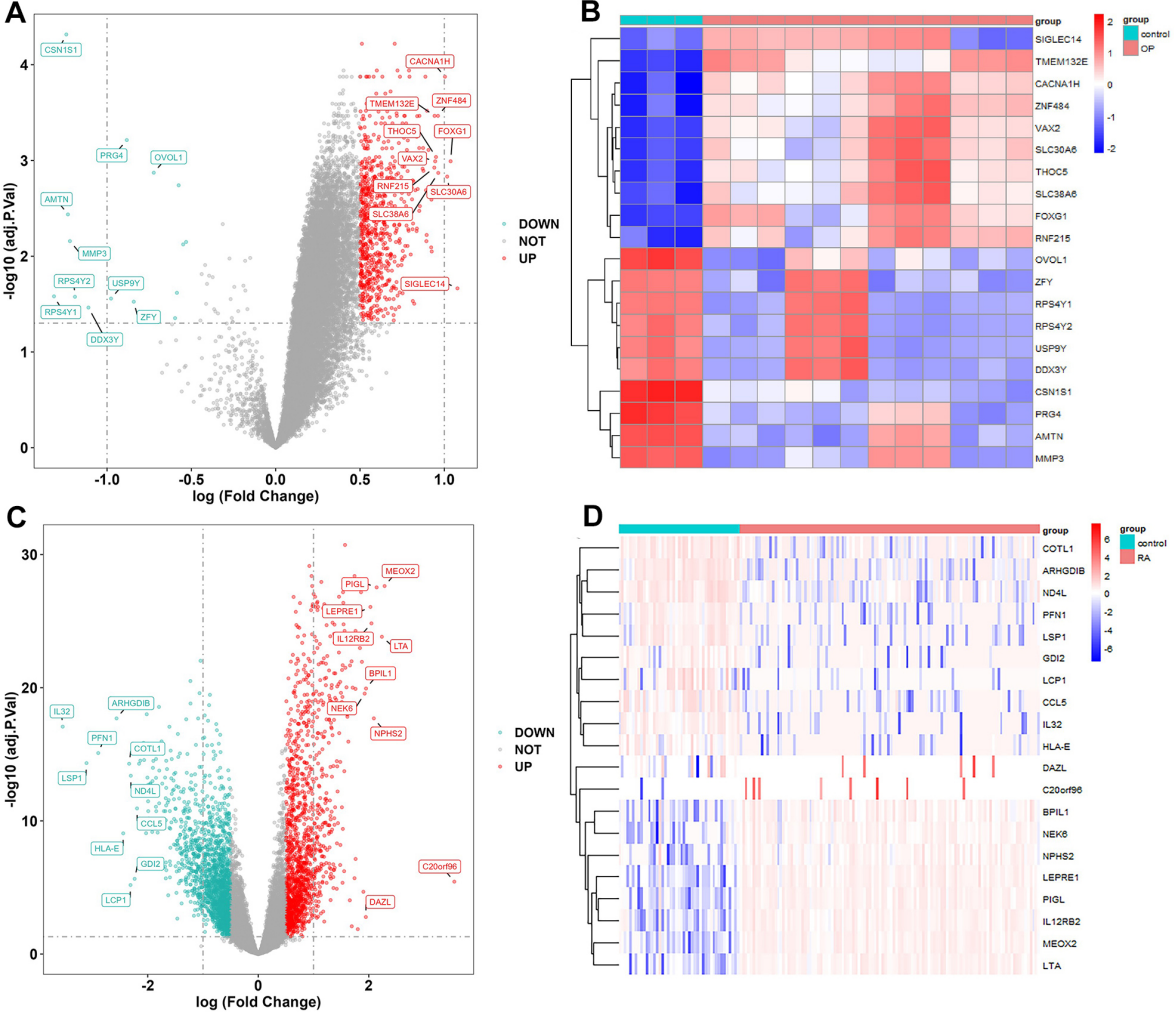
Differential gene expression profiles in OP and (RA). **(A)** volcano plot of DEGs in OP. Red and blue dots represent significantly upregulated and downregulated genes, respectively. **(B)** heatmap of DEGs in OP, demonstrating hierarchical clustering of samples. **(C)** volcano plot of DEGs in RA. **(D)** heatmap of DEGs in RA.

In this study, we used a multi-step approach to identify key genes to ensure that the selected genes were highly associated with RA, OP, and bone metabolism markers. A total of 8589 DEGs between OP and normal groups were screened from the GSE230665 dataset, including 8556 upregulated genes and 33 downregulated genes. The volcano plot and heatmap revealing the clustering relationship between OP and normal group samples are shown in (**Figures 1A, B**), respectively. Next, A total of 3242 DEGs between the RA group and the normal group were screened from the GSE17755 dataset, including 1461 up-regulated genes and 1781 down-regulated genes. The volcano and heat maps revealing the clustering relationship between RA and normal group samples are shown in (**Figures 1C, D**), respectively.

### 3.2 WGCNA and identification of candidate genes

**Figure 2:**
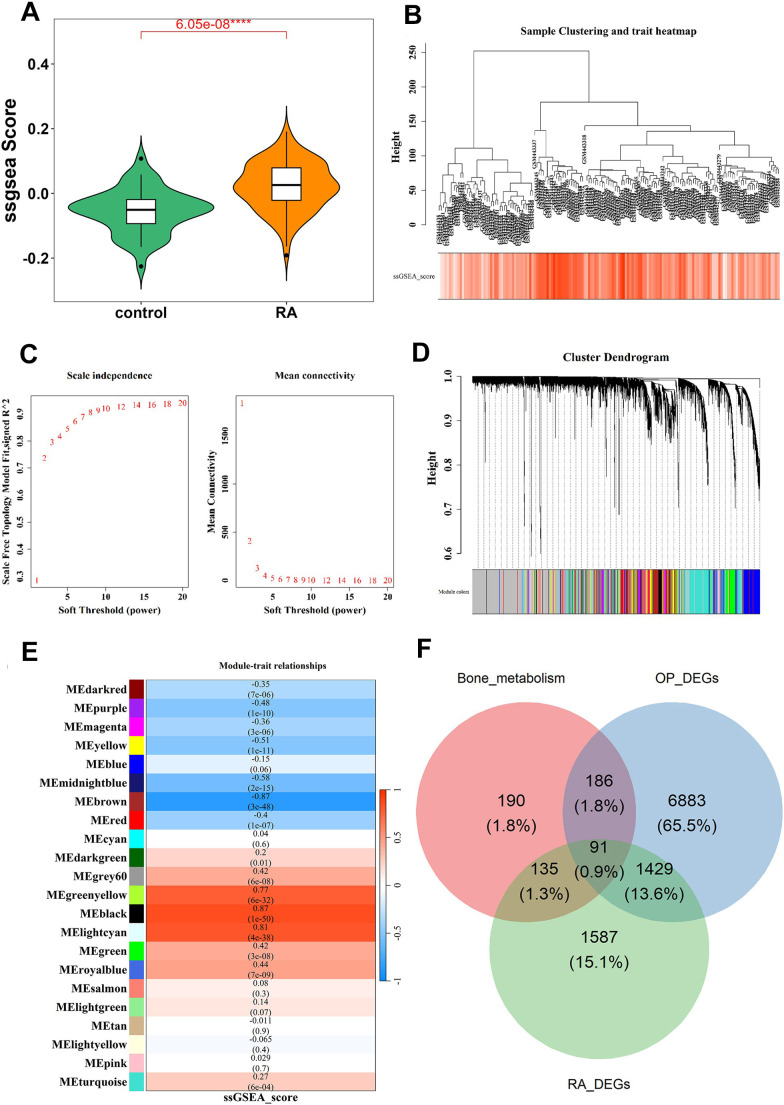
Screening candidate genes using WGCNA analysis and “VennDiagram” software **(A)** conduct ssGSEA scoring on bone metabolism genes to determine inter-group differences; **(B)** perform hierarchical clustering based on the Euclidean distance of sample expression levels to identify outlier samples; **(C)** determine the optimal soft threshold; **(D)** gene co-expression modules, with each color representing a co-expression module; **(E)** heatmap of module-RA associations; the black module shows the strongest correlation; **(F)** candidate genes were identified by Venn diagram of the overlap between differentially expressed genes and bone metabolism genes. (**** P<0.0001).

we performed ssGSEA scoring for bone metabolism-related genes, and the results are shown in (**Figure 2A**), indicating a difference between the two groups. This suggests a correlation between bone metabolism-related genes and RA. Subsequently, we conducted WGCNA analysis based on the scores. Initially, we used Euclidean distance of sample expression levels for hierarchical clustering, and the results showed no outlier samples (**Figure 2B**), allowing us to proceed with subsequent analyses. In our study, we selected a power of β = 8 (scale-free R2 = 0.9) as the soft threshold to ensure a scale-free network (**Figure 2C**). A total of 22 modules were obtained that exhibited similar coexpression patterns among these genes (**Figure 2D**). The module genes in the black module (r=0.87; P = lc-50) were significantly correlated with RA (**Figure 2E**), containing 602 module genes. To identify key genes linking RA and OP through bone metabolism, we cross-analyzed the bone metabolism-related module genes with RA- DEGs and OP-DEGs. This integrative analysis yielded 91 candidate genes, suggesting their putative role in the shared pathogenesis (**Figure 2F**).

### 3.3 Construction of the PPI network and pathway enrichment analysis of hub genes

**Figure 3:**
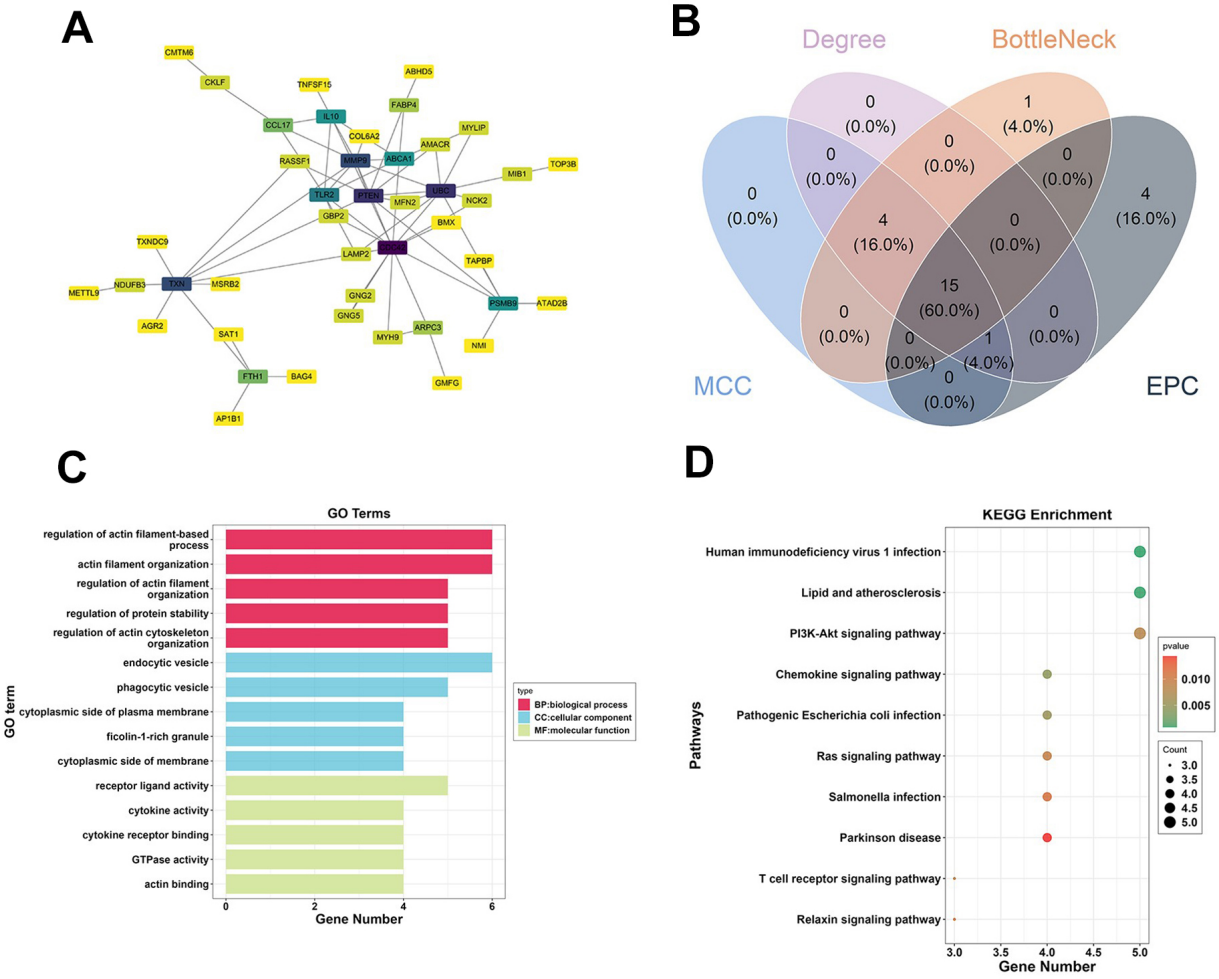
Hub gene selection and enrichment analysis results of key genes **(A)** PPI network diagram; **(B)** four algorithms: ‘Stress’, ‘BottleNeck’, ‘Degree’, and ‘EPC’ are used to screen the core genes in the network, and the intersection of these four algorithms yields 5 hub genes; **(C)** GO analysis of candidate genes; **(D)** KEGG analysis of candidate genes.

A protein-protein interaction (PPI) network was constructed using the 91 candidate genes (**Figure 3A**). Hub genes within this network were identified by applying four algorithms (MCC, BottleNeck, Degree, and EPC). The intersection of the top-ranked genes from each method yielded 15 high-confidence hub genes (**Figure 3B**).

Enrichment analysis was subsequently performed on these 15 hub genes. Gene Ontology (GO) enrichment analysis (p-value < 0.05) returned 490 significant terms, comprising 402 biological processes (BP), 39 cellular components (CC), and 49 molecular functions (MF). The top five most enriched terms in each category, ranked by gene count, are presented (**Figure 3C**). For biological processes (BP), the predominant terms were primarily associated with actin cytoskeleton dynamics and protein stability regulation, including ‘regulation of actin filament-based process’, ‘actin filament organization’, ‘regulation of actin filament organization’, ‘regulation of protein stability’, and ‘regulation of actin cytoskeleton organization’. Within cellular components (CC), the top-ranked terms featured ‘endocytic vesicle’, ‘phagocytic vesicle’, ‘cytoplasmic side of plasma membrane’, ‘ficolin-1-rich granule’, and ‘cytoplasmic side of membrane’. Regarding molecular functions (MF), significant enrichment was observed for ‘receptor ligand activity’, ‘cytokine activity’, ‘cytokine receptor binding’, ‘GTPase activity’, and ‘actin binding’.

KEGG pathway analysis revealed significant enrichment for 147 pathways (p-value < 0.05). The top 10 most enriched pathways are displayed in **Figure 3D**. These included ‘Human immunodeficiency virus 1 infection’, ‘Lipid and atherosclerosis’, ‘PI3K-Akt signaling pathway’, ‘Chemokine signaling pathway’, ‘Pathogenic Escherichia coli infection’, ‘Ras signaling pathway’, ‘Salmonella infection’, ‘Parkinson disease’, ‘T cell receptor signaling pathway’, and the ‘Relaxin signaling pathway’.

### 3.4 Hub gene identification and diagnostic validation

**Figure 4:**
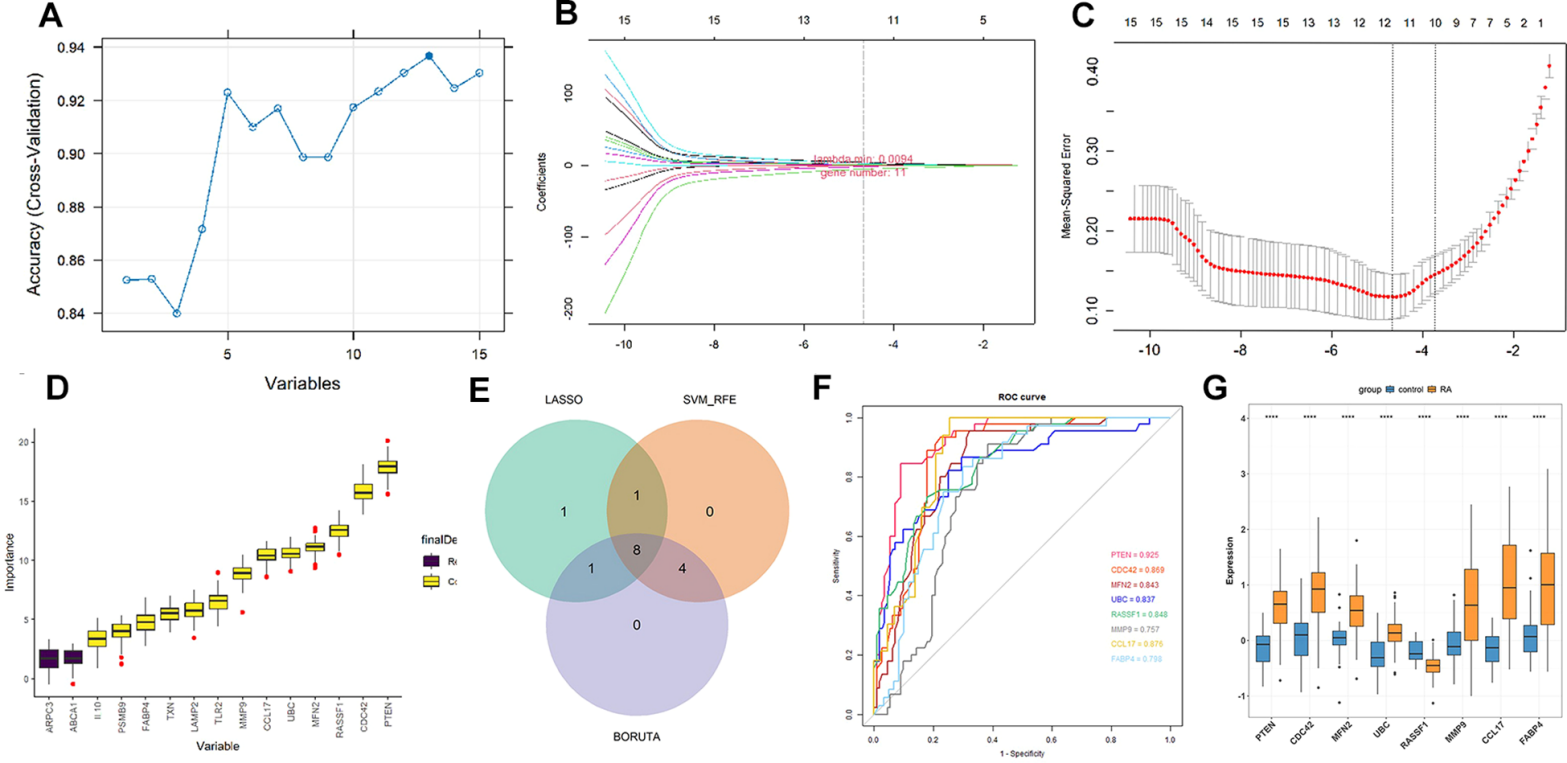
Hub gene identification and diagnostic validation. **(A)** gene subset (n=13) selected by the SVM-RFE algorithm. **(B, C)** LASSO coefficient path **(B)** and 10-fold cross-validation **(C)** for selecting feature genes (n=11). **(D)** feature genes (n=13) confirmed by the Boruta algorithm. **(E)** Venn diagram showing the 8 hub genes derived from the intersection of the three algorithms. **(F)** ROC curves assessing the diagnostic performance of each hub gene. **(G)** expression differences of the hub genes between RA and control groups. Data are presented as box plots. ****P < 0.0001.

When applied to the 15 candidate genes, the SVM-RFE algorithm (with 10-fold cross-validation) identified an optimal feature subset of 13 genes (PTEN, CDC42, RASSF1, MFN2, UBC, TLR2, LAMP2, TXN, PSMB9, ARPC3, MMP9, CCL17, FABP4) at which the highest accuracy was achieved (**Figure 4A**). Simultaneously, LASSO regression analysis pinpointed 11 non-zero coefficient genes (PTEN, CDC42, MFN2, ARPC3, UBC, RASSF1, MMP9, CCL17, ABCA1, FABP4, IL10) at the optimal lambda value (λ = 0.0094) that minimized cross-validation error (**Figures 4B, C**). The Boruta algorithm confirmed 13 decisive features (CCL17, CDC42, FABP4, IL10, LAMP2, MFN2, MMP9, PSMB9, PTEN, RASSF1, TLR2, TXN, UBC) (**Figure 4D**). The intersection of the results from these three algorithms yielded a core set of 8 key genes: PTEN, CDC42, MFN2, UBC, RASSF1, MMP9, CCL17, and FABP4 (**Figure 4E**). ROC curve analysis demonstrated the robust diagnostic potential of these candidate genes, with each exhibiting an Area Under the Curve (AUC) greater than 0.7, indicating good discriminatory capacity (**Figure 4F**). A Wilcoxon rank sum test was conducted to examine for significant differences, and the p-values were all less than 0.0001(**Figure 4G**).

### 3.5 Genomic localization and miRNA prediction of hub genes

**Figure 5:**
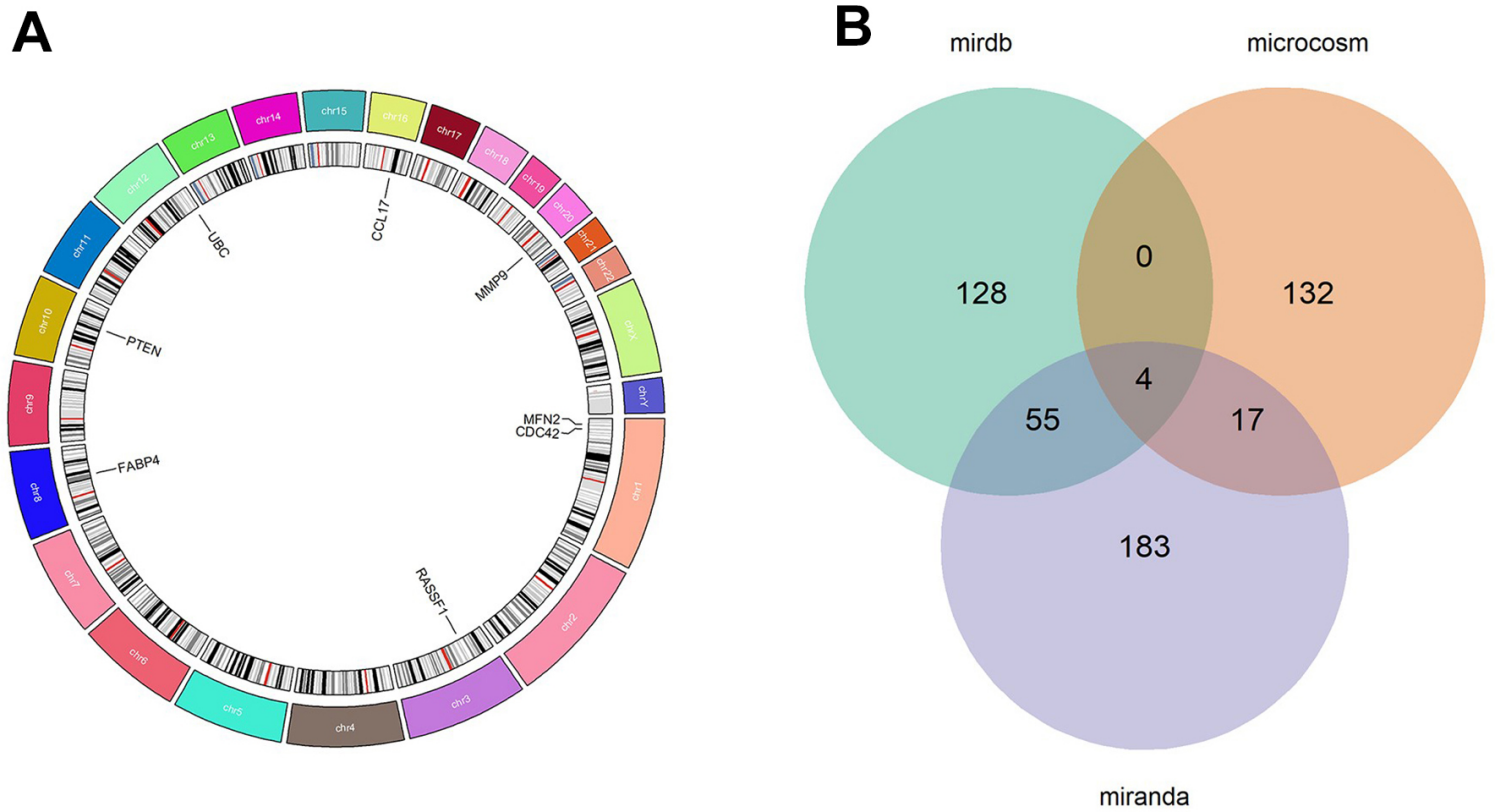
**(A)** map of gene location. **(B)** the predicted miRNAs of the three databases were intersected.

We further characterized the genomic features of the candidate hub genes by determining their chromosomal locations and predicting their regulatory miRNAs (**Figure 5A**). Subsequently, potential miRNAs targeting these key genes were predicted using three databases: miRDB, miRanda, and Microcosm. The intersection of results from all databases identified four high-confidence miRNAs: hsa-miR-185-5p, hsa-miR-499a-3p, hsa-miR-30c-2-3p, and hsa-miR-30c-1-3p. Specifically, FABP4 was predicted to be targeted by hsa-miR-30c-2-3p and hsa-miR-30c-1-3p; CDC42 was associated with hsa-miR-185-5p; and MFN2 was linked to hsa-miR-499a-3p (**Figure 5B**). miRNA prediction is merely exploratory in this study.

### 3.6 Temporal change of weight and joint in CIA rats

**Figure 6:**
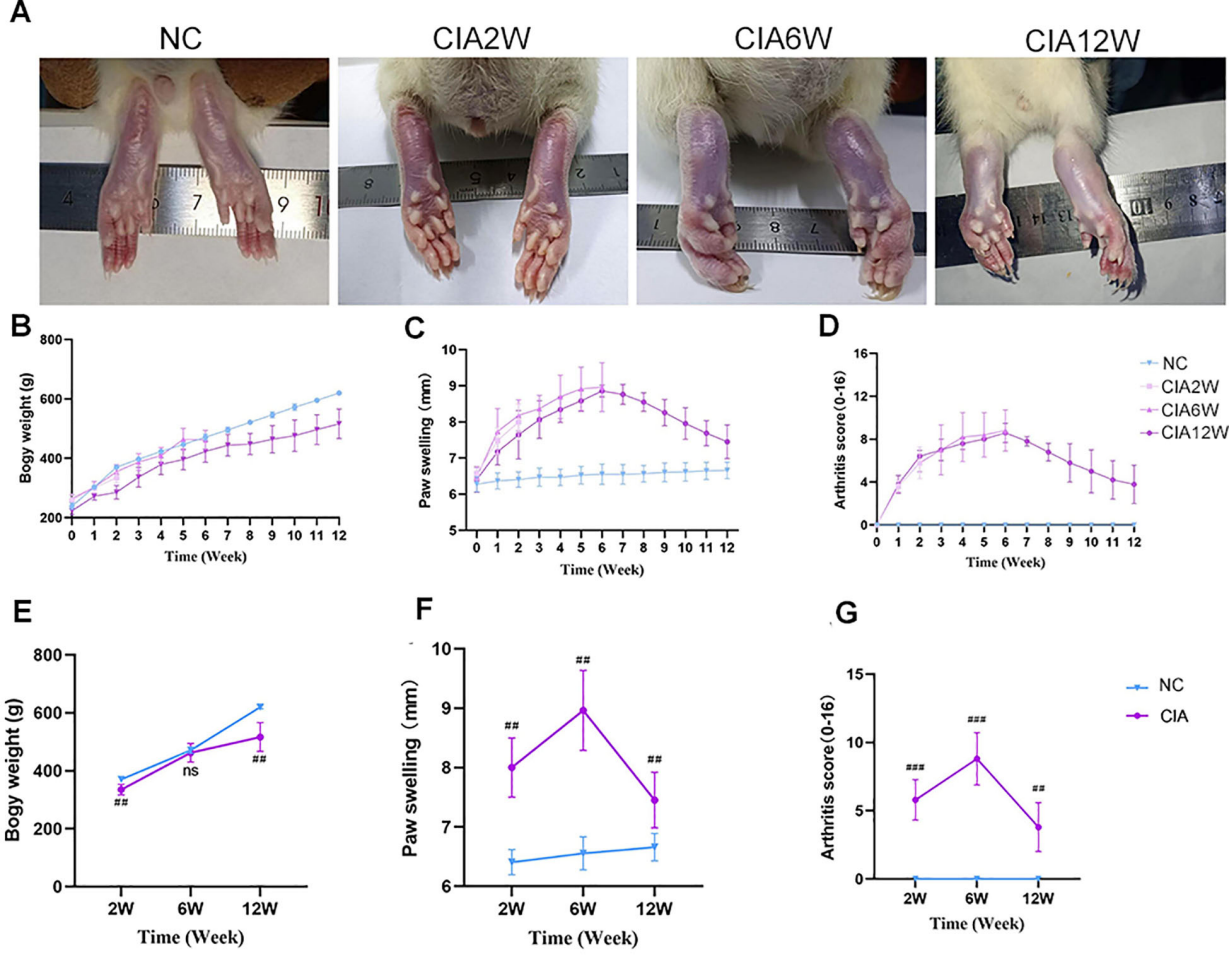
The temporal change of ankle joint in CIA rat. **(A)** representative image of the rat paw showed the redness and swelling. **(B–G)** The observation was initiated after the initial immunization 0 weeks (0w). The body weight, arthritis score, and paw swelling of each group were measured at 0w. Compared to normal control group, ##P<0. 01, ###P<0. 001, ns (non-significant): P>0.05..

The clinical manifestations observed in each CIA model (CIA2W, CIA6W, and CIA12W) demonstrated a marked progression in paw swelling and joint inflammation when compared to the normal control group, with the most severe symptoms occurring at 6 weeks (**Figure 6A**). Relative to the normal control group, the CIA group exhibited more severe slowing of weight gain (**Figure 6B**), paw swelling (**Figure 6C**), and increased arthritis scores (**Figure 6D**), peaking at 6 weeks and subsequently declining slightly by 12 weeks, yet remaining significantly elevated compared to the normal control group throughout the duration of the experiment. Quantitative statistical analyses of body weight, paw swelling, and arthritis scores were conducted for each group. In alignment with morphological observations, paw swelling and arthritis scores in CIA rats showed a significant increase at 2, 6, and 12 weeks, reaching their highest values at 6 weeks, with statistically significant differences observed between the groups (**Figures 6E–G**).

### 3.7 The temporal change of FABP4, inflammatory cytokines, and bone metabolism markers in CIA rats

**Figure 7:**
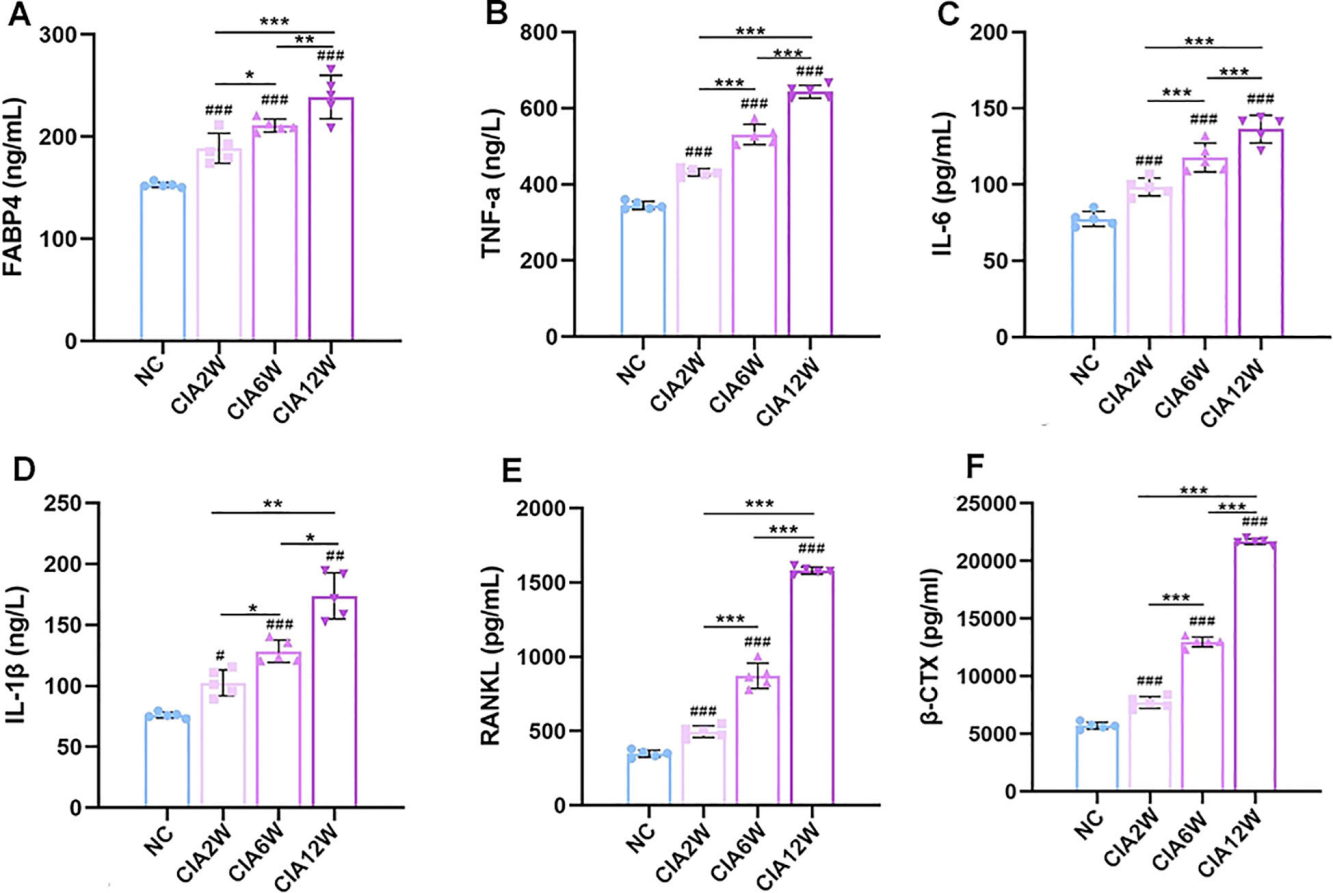
The temporal change of FABP4, inflammatory cytokines, and bone metabolism markers in CIA rats. **(A–F)** the level of FABP4, TNF-a, IL-6, IL-1β, RANKL and β-CTX in serum detected by ELISA. The content of FABP4, TNF-a, IL-6, IL-1β, RANKL and β-CTX was increase at 2 week, 6week and 12week. Compared to normal control group, #P<0. 05, ##P<0. 01, ###P<0. 001. Comparisons were made between different model groups, *P<0. 05, **P<0. 01, ***P<0. 001.

Compared with the normal control group, the levels of FABP4, inflammatory factors TNF-a, IL-6, IL-1β and bone metabolism markers RANKL, β-CTX in the serum of model rats increased at 2w, 6w, 12w (**Figures 7A–F**).

### 3.8 Correlation between FABP4 expression and inflammatory cytokines/bone metabolism markers

**Figure 8:**
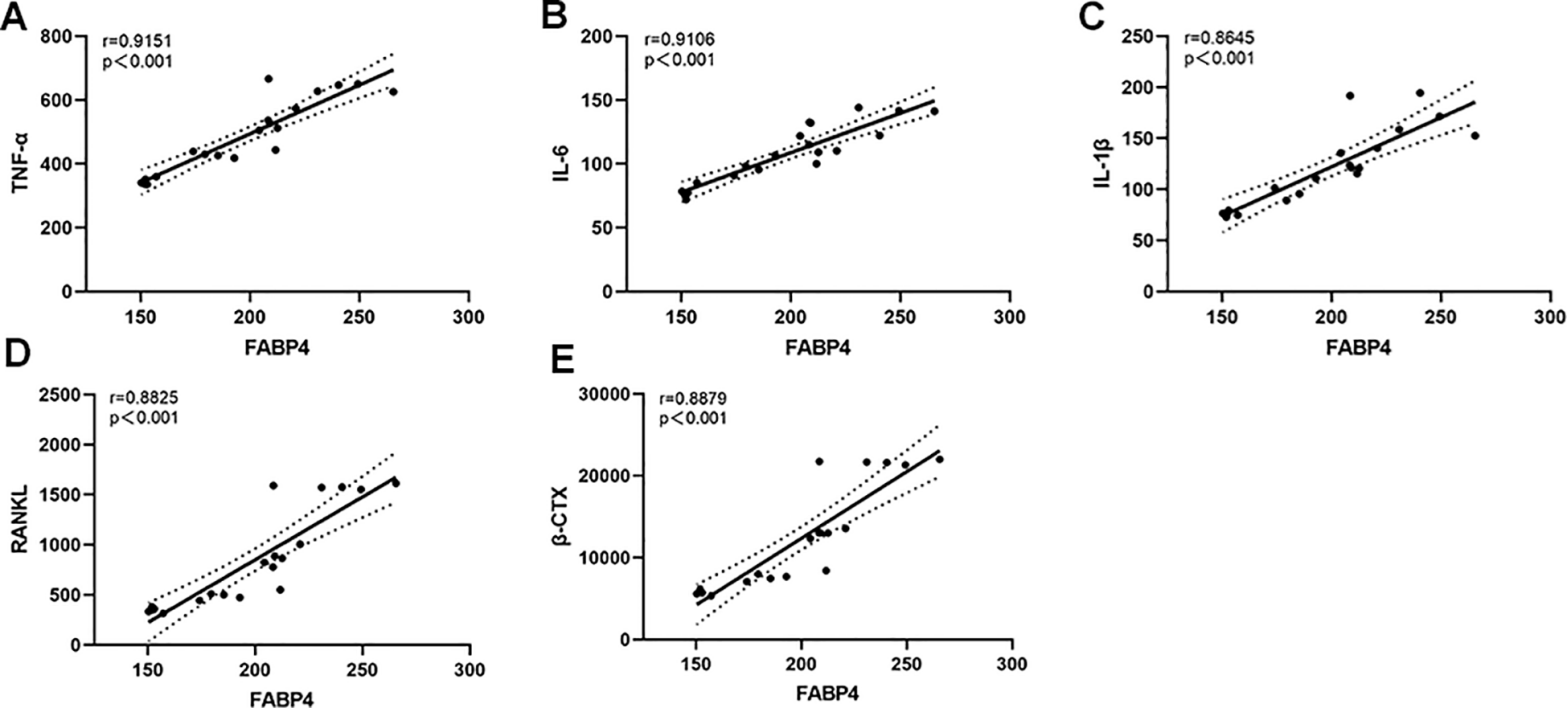
**(A–E)** correlation between FABP4 and IL-1β, IL-6, TNF-α, RANKL, β-CTX.

The results showed that there was a positive correlation between the concentration of FABP4 in rat serum and its inflammatory cytokines TNF-a, IL-6, IL-1βand bone metabolism markers RANKL and β-CTX (**Figures 8A–E**). From the above data, it can be seen that as the inflammatory response in CIA rats intensifies, FABP4 and bone metabolism markers increase positively, indicating that there is a close correlation between FABP4 and bone metabolism markers under inflammatory conditions.

### 3.9 The temporal change of bone destruction in CIA rats

**Figure 9:**
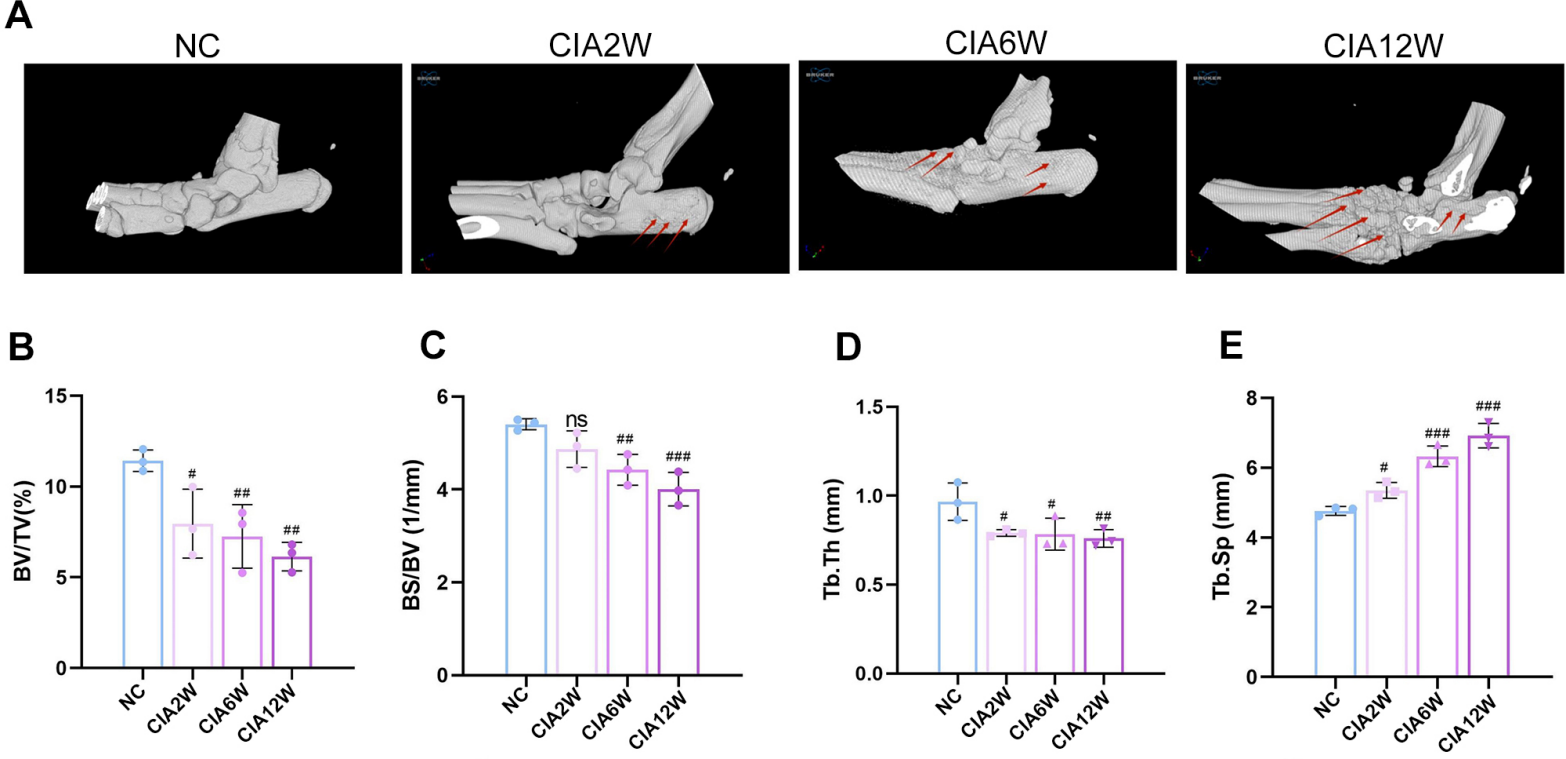
The temporal change of bone destruction in CIA rats. **(A)** representative 3D reconstruction image of paws detected by micro-CT. The area indicated by the red arrow represents the regions of bone erosion, bone defect and bone destruction. **(B–E)** select the region of interest (ROI) within the calcaneal bone block and conduct quantitative statistical analysis of the bone morphometry parameters. BV/TV:bone volume/tissue volume; BS/BV: bone surface/volume; Tb.Th: trabecular thickness; Tb,Sp: trabecular separation #P<0. 05; ##P<0. 01; ###P<0. 001, ns, No significant difference.

Micro-CT imaging revealed that the articular surfaces of rats in all model groups were rough with indistinct joint structures, and bone destruction was progressively aggravated along with disease progression (**Figure 9A**). Compared with the normal control group exhibiting intact bone architecture and continuous, smooth bone cortex, CIA rats at 2 weeks, 6 weeks and 12 weeks presented progressive articular bone destruction. The scope of bone defects expanded continuously in a time-dependent manner, with the most severe bone damage observed at 12 weeks.

Quantitative analysis of calcaneal bone morphometric parameters demonstrated that although no significant difference was detected in bone surface/bone volume (BS/BV) between CIA rats and controls at week 2, bone volume/tissue volume (BV/TV) and trabecular thickness (Tb.Th) were markedly decreased in CIA rats. This descending trend was further exacerbated over time; At weeks 6 and 12 CIA rats, BV/TV, BS/BV, and Tb.Th were markedly lower than in controls (**Figures 9B–D**). Conversely, trabecular separation (Tb.Sp) showed a progressive increase (**Figure 9E**), suggesting ongoing trabecular loss and widening of trabecular spacing, which are characteristic of osteoporosis. These observations need to be confirmed in studies with larger sample sizes.

### 3.10 HE staining of the ankle joint and the expression of FABP4, RANKL and TNF-α in the ankle cartilage

**Figure 10:**
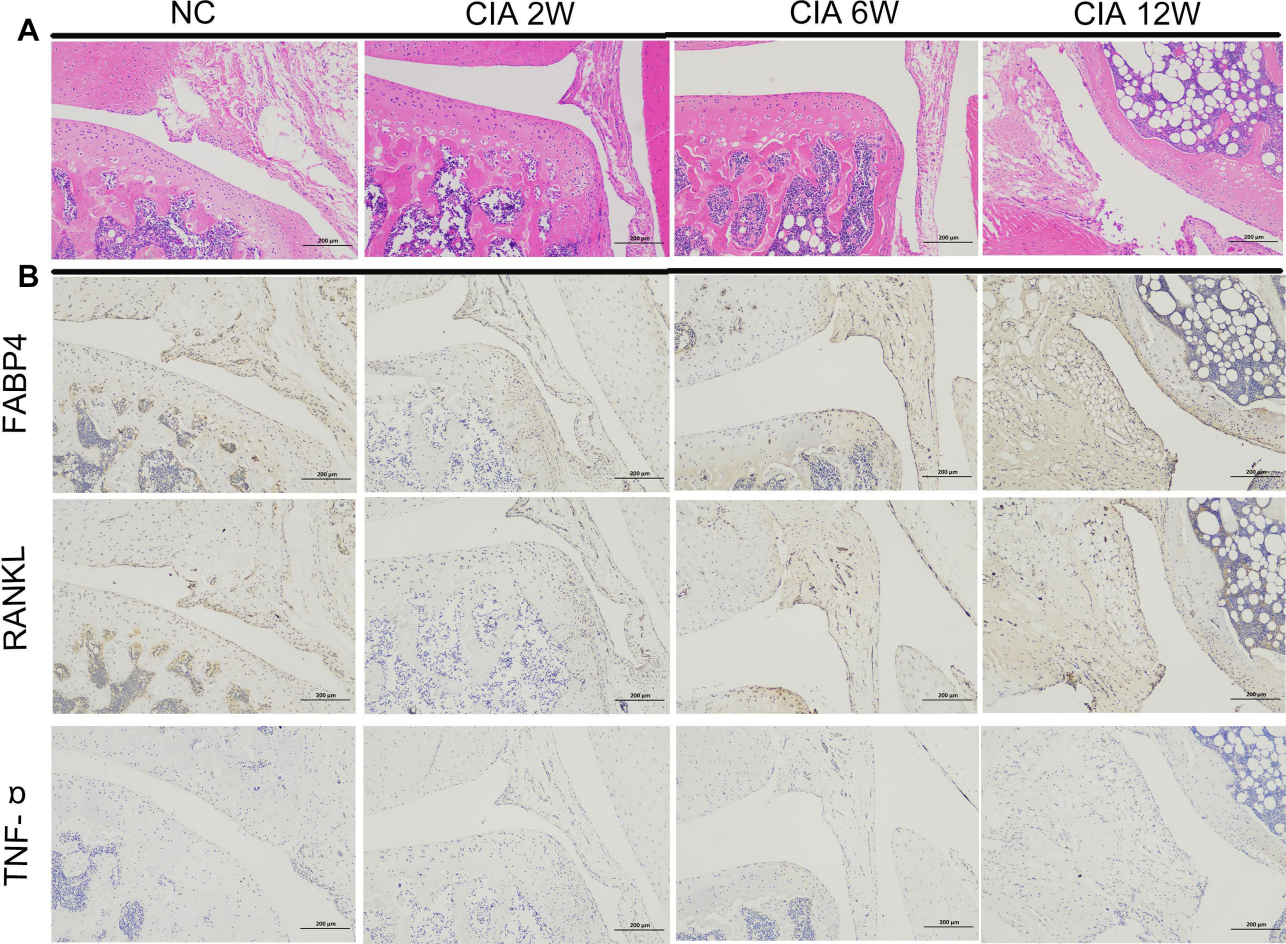
**(A)** Representative pathological sections of the ankle joint using H&E staining (*100). **(B)** Immunohistochemical determination of expression. Representative pictures of FABP4, RANKL, and TNF-a are shown (*100).

H&E staining tissue sections revealed the extent of synovial tissue involvement in the joints of CIA rats (**Figure 10A**). In the tissue sections from the normal control rats, no infiltration of adipocytes was observed. The synovial cells were orderly arranged, the joint surface was smooth and intact, and the chondrocytes exhibited a regular and organized structure. Conversely, CIA rats, two weeks post-immunization, exhibited significant synovial destruction, compromised synovial integrity, disorganized cell arrangement, and adipocyte proliferation. Although the articular cartilage surface showed no overt damage, the chondrocytes were disorganized. After six weeks of immune induction, further deterioration of the synovial membrane integrity was observed, with substantial adipocyte accumulation, disruption of the normal articular cartilage structure, and damage to the cartilage surface. By 12 weeks, the synovial tissue displayed severe disorganization, a large number of cells were fatty, significant erosion of the cartilage surface, loss of normal structural integrity, and a large number of adipocytes were infiltrated.

Immunohistochemical (IHC) analysis revealed that FABP4, TNF-a, and RANKL were positively expressed, as indicated by brown immunostaining, in the synovial tissue of the ankle joint. The normal control group exhibited weak responses of FABP4, TNF-a, and RANKL in synovial and cartilage tissues. In CIA rats, strong expression of FABP4, TNF-a, and RANKL was observed in the synovial and cartilage tissue at 2 weeks. By 6 weeks, the expression of TNF-a began to plateau as the disease progressed, and by 12 weeks, it had nearly returned to normal control levels. Notably, FABP4 expression in both synovium and cartilage peaked at 12 weeks, whereas RANKL expression decreased but remained significantly higher than at 2 weeks (**Figure 10B**).

H-score was performed using ImageJ (**Supplementary Table 1**; **Supplementary Figure 1**). FABP4 H-scores in CIA2W, CIA6W and CIA12W groups were 4.62 ± 1.04, 5.72 ± 0.61 and 9.32 ± 2.23, respectively, all higher than control (0.49 ± 0.17, all P<0.01). FABP4 increased with disease progression; no difference between CIA2W and CIA6W, but CIA12W was higher than CIA6W (P<0.01).

RANKL H-scores at 2, 6, and 12 weeks were 3.76 ± 1.49, 9.81 ± 1.19 and 6.29 ± 0.88, all higher than control (0.55 ± 0.25, all P<0.01). RANKL peaked at 6 weeks (CIA2W vs. CIA6W, P<0.001) and then declined (CIA6W vs. CIA12W, P<0.01) yet remained above the 2week level (P<0.05).

TNF-αexpression exhibited a distinct pattern. TNF-αH-score was elevated in CIA2W (1.03 ± 0.37 vs. control 0.32 ± 0.29, P<0.01), decreased by 6 weeks (0.67 ± 0.01), and returned to near-control levels at 12 weeks (0.27 ± 0.15). These data suggest persistent upregulation of FABP4 and RANKL in CIA joints, whereas TNF-αelevation is mainly early. Given the limited sample size, these findings are preliminary.

## 4 Discussion

Bioinformatics-based GO enrichment analysis revealed that FABP4-associated biological processes were significantly enriched in “actin filament organization,” “regulation of actin cytoskeleton organization,” and “regulation of protein stability.” These findings align with the pathological characteristics of RA FLS, which depend on the rearrangement of the actin cytoskeleton to facilitate migration, invasion, and cartilage erosion (26). Additionally, KEGG pathway enrichment analysis identified FABP4 as being associated with critical pathogenic pathways in RA. The most significantly enriched pathways included the PI3K-Akt signaling pathway, chemokine signaling pathway, Ras signaling pathway, and T cell receptor signaling pathway. The chemokine signaling pathway plays a crucial role in mediating the directional migration of immune cells to the joint synovium, a central event in the early stages of synovitis in RA. The involvement of lipid and atherosclerotic pathways is consistent with the high prevalence of cardiovascular complications in RA patients and the established role of FABP4 as a lipid chaperone involved in metabolic inflammation (27). Among the various factors examined, it is noteworthy that FABP4 is significantly enriched within the PI3K/AKT signaling pathway. FABP4 plays a crucial role in regulating the activation of the PI3K/AKT pathway, thereby promoting the M1 polarization of macrophages and subsequently modulating the inflammatory response (28). The PI3K/AKT signaling pathway is a pivotal regulator of FLS migration and invasion, as well as FLS growth and survival (29). Many experiments evidence has demonstrated that the PI3K/AKT signaling pathway is integral to the formation, differentiation, and survival of osteoclasts (30). It mediates the activation of osteoclasts induced by macrophage colony-stimulating factor (M-CSF), TNF-α, and RANKL, thereby participating in joint inflammation and bone destruction processes (31, 32). Furthermore, the activation of the PI3K/AKT signaling pathway can modulate the OPG/RANKL ratio (33), enhance RANKL expression in osteoblasts, and facilitate osteoclast differentiation (34). The RANKL/RANK/OPG pathway is the central regulatory mechanism in OP secondary to RA. Therefore, it is speculated that FABP4 may influence FLS proliferation via the PI3K/AKT signaling pathway and subsequently regulate the RANKL/RANK/OPG pathway, which is critical in RA-associated OP.

The RANKL/RANK/OPG signaling pathway is extensively acknowledged for its pivotal role in the pathogenesis of secondary osteoporosis associated with rheumatoid arthritis. In this study, PTEN exhibited the highest area under the curve (AUC) value among the eight genes examined (AUC = 0.925). However, our primary interest was directed towards FABP4, which demonstrated significant enrichment in the PI3K/AKT signaling pathway, leading us to focus on FABP4 (AUC = 0.798). A comprehensive literature review revealed a potential link between PTEN and FABP4, suggesting their involvement in rheumatoid arthritis-associated secondary osteoporosis through the PI3K/AKT and RANKL/RANK/OPG pathways. PTEN (phosphatase and tensin homolog deleted on chromosome ten) serves as a negative regulator of the PI3K/Akt pathway, influencing cell cycle regulation, inhibiting cell growth, proliferation, migration, and signal transduction, while promoting apoptosis. The PTEN–PI3K axis modulates the activation of multiple pro-survival signal transduction pathways (35). The loss of PTEN expression can significantly enhance the survival and abnormal proliferation of FLS in rheumatoid arthritis patients, thereby promoting the release of pro-inflammatory cytokines such as IL-1β and TNF-α. Experimental up-regulation of PTEN protein expression has been shown to effectively mitigate joint damage in animal models of rheumatoid arthritis, with its regulatory effects being closely associated with the inhibition of key signaling pathways implicated in the disease (36). A reduction in CDC42 expression is correlated with an elevated risk of rheumatoid arthritis, potentially due to its role in destabilizing T cells, thereby accelerating the autoimmune response, as well as promoting the release of proinflammatory factors such as TNF-α and IL-1β, which exacerbate inflammation and contribute to the pathogenesis and progression of rheumatoid arthritis (37). During aging and in the context of osteoarthritis, MFN2 expression is upregulated. Overexpression of MFN2 has been found to intensify inflammation and joint damage, whereas its knockdown can reverse associated metabolic alterations and ameliorate disease progression (38). *In vitro* studies have demonstrated that silencing the UBC9 gene inhibits the secretion of VEGF-A, MMP-3, and MMP-9 in RA-FLS induced by TNF-α, significantly reducing the proliferation and migratory capacity of FLS. This suggests that UBC9 plays a role in promoting the abnormal activation of RA-FLS, and its inhibition is anticipated to have therapeutic potential. Inhibition of its activity is anticipated to serve as a potential therapeutic target for mitigating the progression of RA (39). FABP4 may be implicated in autophagy through the regulation of the PTEN/Akt/mTOR signaling pathway (40).Therefore, it is concluded that aberrant FABP4 expression in RA may indirectly affect PI3K/AKT pathway activity by affecting lipid metabolism, thereby cross-regulating but not directly interacting with the regulatory role of PTEN. All the above results indicate that the FABP4 target genes screened by bioinformatics analysis in this study are closely related to the pathological process of RA and are valuable for further in-depth study.

Subsequent predictions of microRNAs (miRNAs) targeted by key genes revealed a close association between FABP4 and hsa-miR-30c-2-3p as well as hsa-miR-30c-1-3p. The miR-30 family is involved in the pathogenesis and progression of bone and joint diseases. Zhang et al. (41) demonstrated that miR-30c targets the mRNA of hair nose phalanx syndrome I (Trps1) and can modulate the development of the mesenchymal lineage by selectively inhibiting the differentiation of osteoblasts and chondrocytes, thereby influencing bone development. Another study indicated that the overexpression of miR-30a/b/c/d inhibited BMS2-mediated osteogenic differentiation by targeting Runx2 and Smad1 mRNA, consequently reducing the expression and activity of alkaline phosphatase (42). A comprehensive review article examined the potential involvement of the miR-30 family in the pathogenesis and progression of bone and joint diseases, suggesting that the miR-30 family could serve as a therapeutic target for the diagnosis or treatment of osteoporosis. However, additional *in vivo* experiments are necessary to substantiate its clinical application (43). The evidence also suggests that FABP4-targeted miRNAs might play a role in osteoporosis secondary to RA. In the current study, bioinformatics analyses and animal experiments demonstrated that FABP4 expression levels were elevated and associated with inflammatory cytokines and bone metabolism markers, such as RANKL and β-CTX. Consequently, the modulation of FABP4 by miR-30c may represent an anti-inflammatory mechanism in inflammatory conditions like arthritis, warranting further investigation.

This hypothesis was subsequently tested in rats with CIA. Observations revealed that the ankle joints of CIA rats exhibited swelling and subcutaneous nodule formation at two weeks, mirroring certain clinical features of RA (44). The swelling reached its maximum at 6 weeks and gradually diminished thereafter. The quantitative arthritis score demonstrated a comparable pattern. This limb manifestation can be attributed to the acute onset of inflammation during the early stage, leading to local soft tissue edema, followed by a reduction in local edema as inflammation transitions to a chronic state (45). Notably, serum levels of FABP4 and inflammatory markers (TNF-α, IL-6, IL-1β) in CIA rats continued to rise post-induction, which did not parallel the general appearance trend. This suggests that RA inflammation may not be alleviated and may persist, progressing into chronic inflammation and exhibiting characteristics of OP.

In this study, bone loss in the ankle joint of CIA rats was assessed using Micro-CT. The findings indicated that bone destruction in the ankle joint was mild at 2 weeks, with no significant decrease in BS/BV, confirming that early joint BMD loss was not significantly correlated with sustained inflammation (46). However, both BV/TV and Tb.Th decreased at 2 weeks, indicating that early bone loss may have already commenced. The progressive decline in BS/BV, BV/TV, and Tb.Th observed at weeks 6 and 12 indicates a sustained increase in bone loss, which aligns with the concurrent rise in serum levels of FABP4, RANKL, and CTX. This suggests an ongoing exacerbation of synovial lesions and osteoclast activity. Further research has found that FABP4 is significantly correlated with inflammatory markers RANKL and CTX, suggesting that the secretion of FABP4 by adipocytes and macrophages in hypertrophic synovium increases abnormally, which abnormally promotes the gene transcription and expression of key pro-inflammatory factors such as IL-1β, IL-6, and TNF-α (20), further activates the NF-kB pathway, activates osteoclast activity, and leads to localized bone and cartilage damage, which may be the cause of bone loss.

Histological analyses corroborated persistent synovial and cartilage damage in CIA rats. In the early phase (2 weeks), the synovium exhibited only cellular disarrangement without significant structural abnormalities or evident cartilage damage. Nonetheless, the expression levels of TNF-α and FABP4 were elevated during this period, potentially due to acute inflammatory mediators such as TNF-α, IL-6, and IL-1β, which stimulate T lymphocytes and FLS to release FABP4, thereby exacerbating synovial inflammation (19, 47, 48). At 6 weeks, the elevated expression of FABP4 further amplified the production of inflammatory mediators, intensifying the inflammatory response (49). This hypothesis is corroborated by the observation that the inflammatory scores and macroscopic examination of the CIA rats in our study reached their zenith at 6 weeks. Furthermore, although the visible signs of inflammation in CIA rats diminished after 6 weeks, the sustained elevation of inflammatory markers in the serum suggested that the inflammatory process was not fully resolved. The expression of TNF-α in the synovium and cartilage peaked at 2 weeks and subsequently declined at 6 and 12 weeks, which may reflect a transition from the acute phase of inflammation to a chronic inflammatory state. The levels of inflammatory markers in peripheral blood exhibited a continuous increase, a trend not mirrored by the levels observed in the synovium and cartilage. This discrepancy is hypothesized to arise from inherent differences between blood and synovial tissue, warranting further investigation. Chronic inflammation has the potential to cause sustained damage to the synovium and cartilage, which may contribute to the localized bone destruction associated with RA (50). Research on persistent inflammation indicates that a deficiency in FABP4 can directly inhibit the activation of NF-κB, suppress the MAPK pathway, and reduce osteoclast formation activity (23). Proinflammatory cytokines regulated by FABP4, such as IL-1β, TNF-α, and IL-6, can target synovial fibroblasts, synoviocytes, and T cells, subsequently inducing RANKL expression through NF-κB activation (51). In the current study, histological and immunohistochemical analyses revealed that FABP4 overexpression in RA may modulate the RANKL/RANK/OPG signaling pathway, thereby exacerbating local bone loss. This study integrates bioinformatics analysis with animal experiments to provide pertinent evidence indicating a close association between FABP4 and abnormal bone metabolism mediated by the RANKL/RANK/OPG system. However, it is important to acknowledge that causality has not been definitively established. The findings suggest that FABP4 may influence the pathological phenotype of FLSs through the RANKL/RANK/OPG system by modulating the PI3K/AKT signaling pathway and inflammatory response, thereby potentially exacerbating osteoporosis in RA.

In conclusion, this study proposes that the FABP4 gene may contribute to bone destruction mediated by the RANKL/RANK/OPG system in secondary osteoporosis associated with RA. Furthermore, the study confirms the specific expression of FABP4 in serum and synovial and cartilage tissue of CIA rats, noting a significant upregulation of FABP4 expression as the disease progresses. Nonetheless, direct causality has not been confirmed, and the role of FABP4 requires further validation through functional experiments, such as FABP4 knockdown or overexpression studies. These exploratory findings may enhance our comprehension of the progression of secondary osteoporosis in RA and support the identification of potential prognostic biomarkers and therapeutic targets. However, our study has several limitations. Firstly, the causal role of FABP4 has not been validated through cellular experiments, such as observing osteoclast differentiation following FABP4 knockdown, or through animal models, such as evaluating bone mineral density in FABP4 knockout mice. Secondly, we did not conduct pathway blockade experiments, such as using PI3K/AKT inhibitors, to directly test the proposed mechanism. Thirdly, the sample size in the CIA model was limited (n = 5 per group), rendering the proposed mechanism speculative and necessitating validation through direct experimentation. Fourthly, the experimental design did not incorporate an independent osteoporosis model group, which constrains the specificity of our hypothesis concerning secondary osteoporosis in RA. Our research remains in its early stages. Future studies will focus on experimental investigations involving FABP4 inhibitors, promoters, and gene knockout, in addition to increasing the number of osteoporosis control groups, to address the current limitations.

## Data Availability

The datasets presented in this study can be found in online repositories. The names of the repository/repositories and accession number(s) can be found in the article/**Supplementary Material**.
