# Supplementary figures and images for: The potential involvement of FABP4 in rheumatoid arthritis-associated osteoporosis: a comprehensive bioinformatics analysis and experimental validation
Source: Front Immunol. 2026 May 28;17:1738443. doi: 10.3389/fimmu.2026.1738443 (PMC13254189; doi:10.3389/fimmu.2026.1738443)

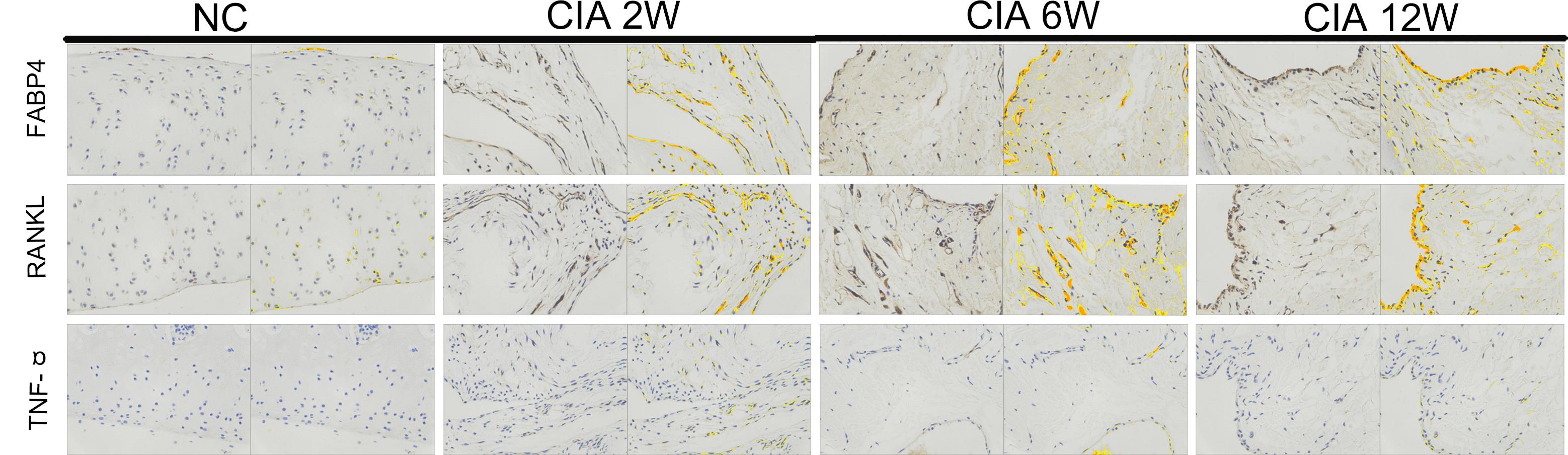

Supplement: Supplementary file 1 [file SupplementaryFile1.jpg]

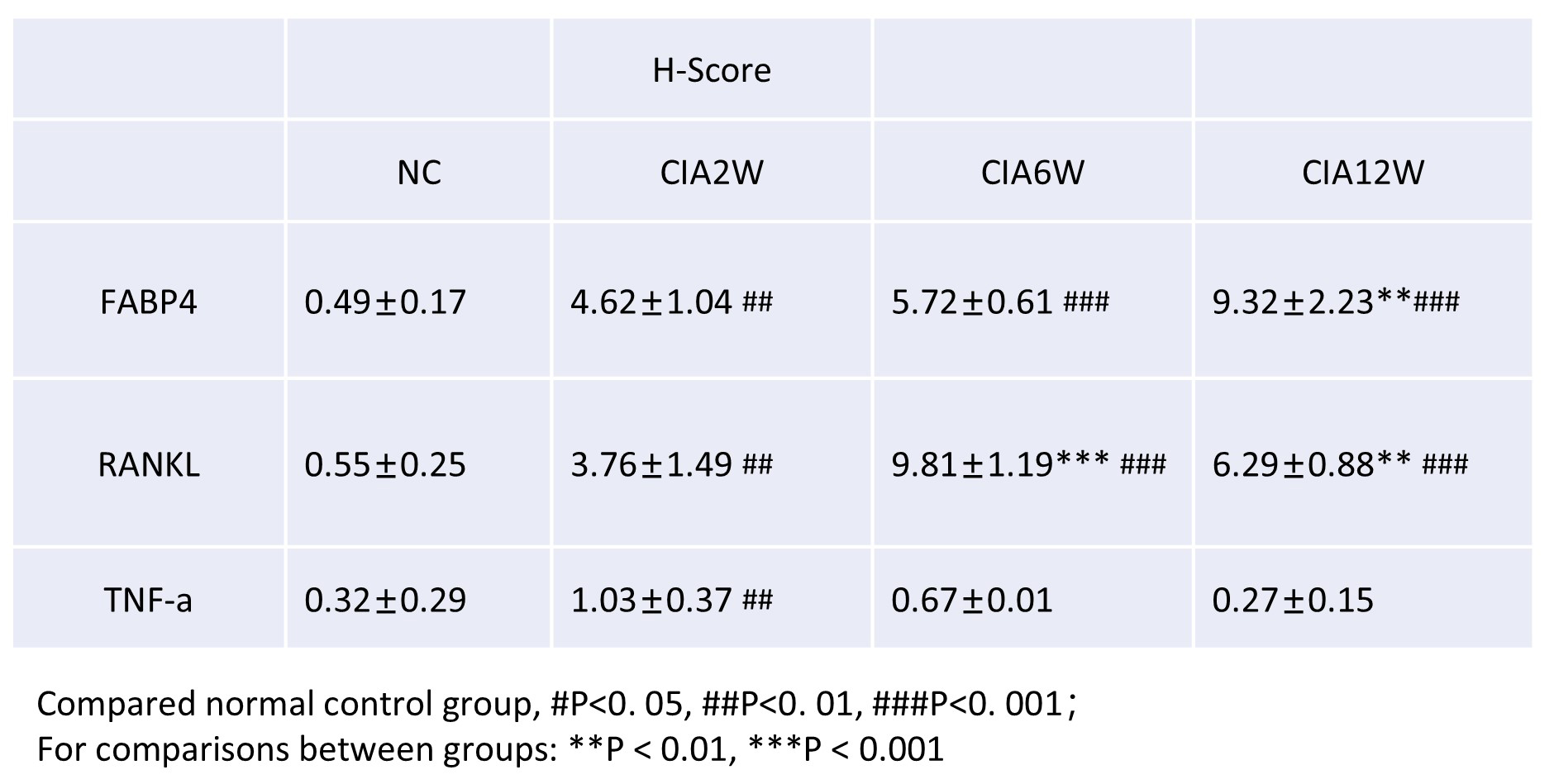

Supplement: Supplementary file 2 [file SupplementaryFile2.jpg]
